# CMR detects decreased myocardial deformation in asymptomatic patients at risk for heart failure

**DOI:** 10.3389/fcvm.2022.1091768

**Published:** 2023-01-05

**Authors:** Djawid Hashemi, Patrick Doeblin, Moritz Blum, Karl Jakob Weiss, Matthias Schneider, Grigorios Korosoglou, Rebecca Elisabeth Beyer, Burkert Pieske, Frank Edelmann, Sebastian Kelle

**Affiliations:** ^1^Department of Internal Medicine and Cardiology, Charité – Universitätsmedizin Berlin, Charité Campus Virchow Clinic, Berlin, Germany; ^2^Department of Internal Medicine and Cardiology, German Heart Institute Berlin (DHZB), Berlin, Germany; ^3^DZHK (German Centre for Cardiovascular Research), Partner Site Berlin, Berlin, Germany; ^4^BIH Charité Digital Clinician Scientist Program, Berlin Institute of Health at Charité – Universitätsmedizin Berlin, BIH Biomedical Innovation Academy, Berlin, Germany; ^5^Brookdale Department of Geriatrics and Palliative Medicine, Icahn School of Medicine at Mount Sinai, New York, NY, United States; ^6^Department of Cardiology, Vascular Medicine and Pneumology, Gesundheitszentrum Rhein-Neckar Hospital Weinheim, Weinheim, Germany; ^7^Cardiac Imaging Center Weinheim, Hector Foundation, Weinheim, Germany

**Keywords:** heart failure, cardiovascular magnetic resonance imaging, myocardial deformation, strain, handgrip exercise, risk, asymptomatic

## Abstract

**Aims:**

The main management strategy of heart failure with preserved ejection fraction (HFpEF) is prevention since HFpEF is associated with many cardiovascular (CV) risk factors, especially since HFpEF is linked to a high risk for both mortality and recurrent heart failure (HF) hospitalizations. Therefore, there is a need for new tools to identify patients with a high risk profile early. Regional strain assessment by CMR seems to be superior in describing deformation impairment in HF. The MyoHealth score is a promising tool to identify cardiac changes early.

**Methods and results:**

Heart failure patients irrespective of LVEF and asymptomatic controls were recruited, and CMR based measures were obtained. For this analysis the asymptomatic control group (*n* = 19) was divided into asymptomatic subjects without CV co-morbidities or evidence of cardiac abnormalities and (*n* = 12) and asymptomatic subjects with CV co-morbidities or evidence of cardiac abnormalities (*n* = 7) as well as patients with HFpEF (*n* = 19). We performed CMR scans at rest and during a stress test using isometric handgrip exercise (HG). Assessing the MyoHealth score at rest revealed preserved regional strain in 85 ± 9% of LV segments in controls, 73 ± 11% in at Risk subjects and 73 ± 8% in HFpEF patients. During stress the MyoHealth score was 84 ± 7% in controls, 83 ± 7 in at risk subjects and 74 ± 11 in HFpEF patients.

**Conclusion:**

In summary, we show for the first time that asymptomatic subjects with increased CV risk present with HFpEF like impaired myocardial deformation at rest, while they show results like controls under HG stress. The potential of preventive treatment in this group of patients merits further investigation in future.

**Clinical trial registration:**

[https://drks.de/search/de/trial/DRKS00015615], identifier [DRKS00015615].

## 1. Introduction

Heart failure with preserved ejection fraction (HFpEF) is defined as symptomatic heart failure (HF), a left ventricular ejection fraction (LVEF) ≥ 50% and evidence of diastolic dysfunction and/or raised LV filling pressures (1). HFpEF is associated with a variety of cardiovascular (CV) risk factors and a high risk for both mortality and recurrent HF hospitalization (2, 3).

With very limited and only recently introduced treatment options, prevention including the early identification of vulnerable patients with CV risk factors, remains the focus of HFpEF management (4, 5). This challenge is intensified by the increasing prevalence of HFpEF, triggered by the lower mortality of cardiovascular risk factors, e.g., diabetes or arterial hypertension (6, 7). Hence, the Universal Definition and Classification of HF considers myocardial changes in still asymptomatic patients already as Stage A and B of HF and encourages earlier action to prevent a transition in clinical apparent HF (8).

Once, HFpEF is suspected, the introduced algorithm to diagnose HFpEF is a comprehensive approach requiring multiple steps (2). The more pronounced the characteristic details of HFpEF are, the worse is the patients’ prognosis (3). It has been shown that patients at risk for HFpEF have already an increased mortality and risk for HF hospitalization (3). The scarcity of resources forces health care providers to identify patients at risk to optimize their therapy continuously.

Hence, there is an urgent need to screen for patients at risk in clinical routine to prevent HFpEF.

Cardiovascular magnetic resonance imaging (CMR) provides anatomical and functional cardiac parameters. Myocardial strain analysis may detect impaired myocardial contractility in patients despite a preserved LVEF (9). Regional strain assessment seems to be superior to global strain analysis in describing deformation impairment in HF (10). The MyoHealth score reflects the share of LV segments with preserved strain values (≤ −17%) in a 37 segment model (10).

We hypothesize that asymptomatic healthy subjects with CV co-morbidities may demonstrate detectable impairments in regional strain compatible with pre-clinical HFpEF.

## 2. Method

This study was a prospective study conducted in Berlin, Germany, approved by the local Ethics Committee (registration: EA4/112/16; German Clinical Trials Registry, DRKS, DRKS00015615). Its rationale and design have been described previously (10–13).

Briefly, HF patients irrespective of LVEF and controls without HF were recruited, and CMR based measures of cardiac structure and function, including assessment of cardiac contractility were obtained. For this analysis, we included the control subjects and the HFpEF patients.

We divided the control group into (a) subjects without HF and no CV co-morbidities or evidence of cardiac dysfunction and (b) those without HF but CV co-morbidities or evidence of cardiac dysfunction. CV co-morbidities or evidence of cardiac dysfunction were defined as the presence of diabetes, suboptimal managed arterial hypertension (hypertensive values at rest despite medication), increased NT-proBNP levels (>120 pg/dL), or LV hypertrophy (LV wall thickness > 11 mm) on CMR (minimum 1 criterion). They were compared to (c) patients with HFpEF (10). We performed CMR scans at rest and during a non-invasive, medication-free stress test. For stress testing we used isometric handgrip exercise (HG), which was effective and changed both blood pressure and heart rate significantly (11). All patients were in sinus rhythm, *nota bene* patients with atrial fibrillation were excluded to maintain better CMR image quality.

All CMR images were acquired using 1.5 T, fast strain-encoded MRI was used for strain evaluation. Volume measurements were performed with Medis^®^ Suite MR (Medis medical imaging systems, Leiden, The Netherlands, version 3.1), strain analysis by MyoStrain (Myocardial Solutions, Inc., Morrisville, North Carolina, USA, version 5.2) (10, 11).

Pairwise comparisons were conducted using a student’s *t*-test, comparisons across three groups were conducted using one-way analysis of variance (one-way ANOVA). A *P*-value < 0.05 was considered statistically significant.

The endpoint was the MyoHealth score at rest and under HG.

## 3. Results

The baseline characteristics of the three groups (controls without CV risk factors: *n* = 12; controls with CV risk factors: *n* = 7 and HFpEF: *n* = 19) are shown in Table 1. The LVEF was similar in all three cohorts: LVEF median [IQR; Q_1_–Q_3_]: control: 63.00 [59.22–64.70]%; at risk: 61.52 [57.88–64.58]%; HFpEF: 61.12 [58.17–64.17]%.

**TABLE 1:** Baseline characteristics.

Parameters	Controls (*n* = 12)	At risk (*n* = 7)	HFpEF (*n* = 19)	*P*-value
Age – median [IQR], years	59.00 [54.75–65.00]	67.00 [62.00–72.00]	78.00 [75.00–82.00]	<0.01*
Female sex – no. (%)	6 (50.00)	4 (57.14)	9 (47.37)	0.914
LVEF – median [IQR], %	63.00 [59.22–64.70]	61.52 [57.88–64.58]	61.12 [58.17–64.17]	0.888
LA area – median [IQR], cm^2^	20.00 [16.50–22.75]	21.00 [19.00–21.00]	22.50 [16.75–25.00]	0.73
NT-proBNP – median [IQR]), pg/dL	66.00 [50.50–88.00]	114.00 [58.50–175.50]	314.00 [266.00–617.00]	<0.01*
hs-TroponinT – median [IQR], (ng/l)	6.00 [4.00–7.50]	8.00 [5.5–10.00]	13.50 [9.00–20.00]	0.02*
eGFR – median [IQR]), mL/min/1.73 m^2^	86.00 [71.50–90.00]	82.00 [72.50–85.50]	74.00 [60.25–82.75]	0.06
Coronary artery disease – no. (%)	0 (0)	0 (0)	12 (66.67)	<0.01*
Arterial hypertension [*n* (%)]	4 (33.33)	3 (42.86)	17 (89.47)	<0.01*
Diabetes – no. (%)	0 (0)	3.00 (42.86)	7.00 (36.84)	0.04*
Dyslipidemia – no. (%)	2.00 (16.67)	2.00 (28.57)	12 (66.67)	0.02*

eGFR, estimated glomerular filtration rate; IQR, interquartile range (first quartile – third quartile); LV, left ventricular; LVEF, left ventricular ejection fraction; N, number. *Statistically significant. Further details on the baseline characteristics described by Blum et al. (11).

**FIGURE 1:**
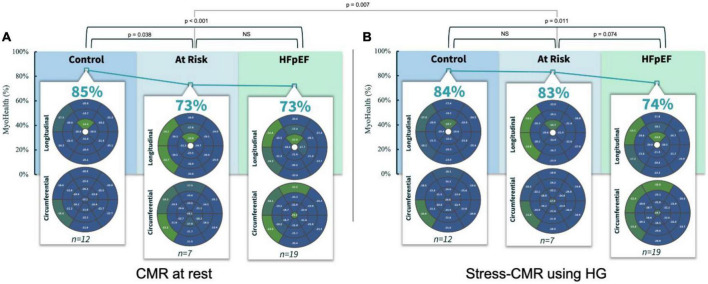
MyoHealth score at rest **(A)** and under stress **(B)** in healthy controls, controls with CV risk factors and HFpEF patients. CMR, cardiovascular magnetic resonance imaging; HG, handgrip exercise; NS, statistically not significant. Preserved regional strain values are blue, altered regional strain values are green. Color code reflects that early affected regions are primarily septal segments. In the at risk group the altered septal segments improve during HG stress, reflected by a color change from green to blue.

Assessing the MyoHealth score at rest revealed preserved regional strain in 85 ± 9% of LV segments in controls, 73 ± 11% in at risk subjects and 73 ± 8% in HFpEF patients (comparisons in Figure 1A and Table 2). During stress the MyoHealth score was 84 ± 7% in controls, 83 ± 7 in at Risk subjects and 74 ± 11 in HFpEF patients (comparisons in Figure 1B and Table 2).

**TABLE 2:** Comparison of the MyoHealth results.

	MyoHealth values at rest	MyoHealth values during HG stress	*P*-value, student’s *t*-test
Controls –%, median [IQR]	86.49 [82.77–89.19]	86.49 [78.14–87.51]	0.71
At risk –%, median [IQR]	73.33 [69.82–78.38]	83.33 [79.50–87.08]	0.01
HFpEF –%, median [IQR]	72.97 [68.92–78.38]	72.97 [70.27–78.38]	1.00
*P*-value, one-way ANOVA	<0.01	<0.01	

ANOVA, analysis of variance; HG, handgrip exercise; IQR, interquartile range.

At rest, the MyoHealth score in at the risk cohort was reduced compared to the healthy controls (*p* = 0.04), at the same level as the HFpEF cohort (*p* = 0.45). This is in line with our recent finding that demonstrated the potential diagnostic window across different heart-failure stages using CMR-strain-analysis (14). However, during stress, at the risk cohort showed a higher MyoHealth score and was similar to the healthy controls (*p* = 0.32) and higher than the HFpEF values (*p* = 0.07). The “at risk” group improved significantly between rest and stress (*p* = 0.01, Figures 1A, B), while there were no relevant changes in healthy controls (*p* = 0.36) or HFpEF (*p* = 0.35). Like the HFpEF pattern, the impaired segments were mainly septal. During stress the impaired septal segments improved primarily in terms of circumferential strain (Figure 1B). It has been shown that septal impairment precedes global systolic dysfunction, highlighting the relevance of septal assessments in the future (10).

## 4. Discussion

In summary, we show for the first time that asymptomatic subjects with evidence of CV risk present with HFpEF like impaired myocardial deformation at rest. The absence of HF symptoms in these subjects is well explained by the compensation capacities during stress when their deformation capacities are similar to healthy subjects.

Performing a quick medication-free CMR-stress-test as HG in asymptomatic patients provides the chance to assess cardiac manifestations of their individual risk-profile. In patients with pathological changes, a stricter management of co-morbidities and shorter follow-up intervals may be adequate to prevent the transition to HFpEF. The potential of preventive treatment in this group of patients merits further investigation in future studies. The feasibility of its use in clinical practice is underlined by the quick acquisition of the exam as it added only up to 10 min. to the regular scan protocol during our study. This time included the more extensive informed consent process regarding the HG application and the additional image acquisition during the HG test.

Table 1 shows an age difference between the three groups, the difference between the youngest, the control subjects, and the HFpEF group was 19 years. This finding might suggest that the results reflect changes in elderly constitutions also supported by higher NT-proBNP values in the older group (15). However, we believe that our findings reflect different disease stages which are also influenced by age, but the main age-depended factor influencing both serum biomarkers and cardiac constitutions is atrial fibrillation which was excluded while recruiting the subjects. Therefore, we see the data has representative for theoretical patient trajectory from healthy to suffering from HF (16).

However, the limited number of subjects included in this study restrains the generalizability of the results. Nonetheless, the reasoning that changes in cardiac function do not develop at a certain tipping point but are present to some degree even at a preclinical state is both shown and intuitive – we propose an emerging tool promising to detect changes early.

Therefore, CMR scans including HG are a promising tool in future preventive cardiology trials for better risk stratification and phenotyping.

## Data availability statement

The datasets presented in this article are not readily available because data safety regulations of the informed consent process limit open access. Requests to access the datasets should be directed to DH, djawid.hashemi@charite.de.

## Ethics statement

The studies involving human participants were reviewed and approved by the Charité – Universitätsmedizin Berlin Ethics Committee. The patients/participants provided their written informed consent to participate in this study.

## Author contributions

DH, FE, and SK: conception and design of the study and literature review. DH, MB, and SK: analysis and interpretation of the data. DH: drafting of the manuscript. DH, PD, and MB: data collection. All authors contributed to revising and editing the manuscript.
